# Supplementary material for: Is Loneliness an Undervalued Pathway between Socio-Economic Disadvantage and Health?
Source: Int J Environ Res Public Health. 2021 Sep 28;18(19):10177. doi: 10.3390/ijerph181910177 (PMC8508269; doi:10.3390/ijerph181910177)
Supplement: Supplementary file 1 [file ijerph-18-10177-s001.zip › ijerph-1293325-supplementary.pdf]

Supplementary Materials

Table S1. Categories, operationalization, and sources of dependent and independent variables.

Variable	Category	Coded	Source	Variable	Category	Coded	Source
**Age**	19-40	0	Statistics Netherlands	**Physical activity**	Insufficient	1	Health survey
	41-64	1	Statistics Netherlands		Sufficient	0	Health survey
	65-80	2	Statistics Netherlands	**Body Mass Index (BMI)**	Underweight (<18,5)	1	Health survey
	81+	3	Statistics Netherlands		Normal (18,5-25)	0	Health survey
**Sex**	male	0	Statistics Netherlands		Overweight (25-30)	2	Health survey
	female	1	Statistics Netherlands		Obese (30>)	3	Health survey
**Migration status**	Dutch born	0	Statistics Netherlands	**Alcohol consumption**	Never	0	Health survey
	Western migration background	1	Statistics Netherlands		Regular consumption	1	Health survey
	Non-western migration background	2	Statistics Netherlands		Excessive	2	Health survey
**Marital status**	married or living together	0	Health survey	**Smoking**	Never smoked	0	Health survey
	single	1	Health survey		Former smoker	1	Health survey
	divorced	2	Health survey		Current smoker	2	Health survey
	widowed	3	Health survey	**Loneliness**	Score 0-11	n/a	Health survey
**Education**	Primary school	3	Health survey	**Chronic disease**	None	0	Health survey
	Lower vocational	2	Health survey		At least one	1	Health survey
	Middle vocational/ secondary	1	Health survey	**Self-rated health**	(very) good, excellent	0	Health survey
	Higher vocational/ university	0	Health survey		fair, bad	1	Health survey
**Household income quartile**	0-25%	3	Statistics Netherlands	**Psychological distress**	No or low risk (score 10-29)	0	Health survey
	26-50%	2	Statistics Netherlands		High risk (score 30-50)	1	Health survey
	51-75%	1	Statistics Netherlands	**Mode of survey completion**	Paper	0	Health survey
	76-100%	0	Statistics Netherlands		Internet	1	Health survey
**Self-reported income adequacy**	Inadequate, major concerns	3	Health survey		Face-to-face interview	2	Health survey
	Inadequate, some concerns	2	Health survey		Telephone interview	3	Health survey
	Adequate, minor concerns	1	Health survey				
	Adequate, no concerns	0	Health survey				

Table S2. Goodness-of-fit tests per health outcome and model.

Health outcome	Goodness-of-fit test	Model 1		Model 2		Model 3		Model 4	
		F	p-value	F	p-value	F	p-value	F	p-value
**Chronic disease**	**Deviance**	280641	1.00	243286.9	1.00	271587.1	1.00	231737.3	1.00
	**Pearson**	242381.1	1.00	210064.2	1.00	237570.5	1.00	206915.2	1.00
**Self-rated health**	**Deviance**	256432.6	1.00	215575	1.00	239907.2	1.00	199533.8	1.00
	**Pearson**	289834.5	1.00	252750.8	1.00	280249	1.00	242259.9	1.00
**Psychological distress**	**Pearson**	0.58	0.82	0.36	0.95	10.42	0.00*	7.80	0.00*

*goodness of fit statistics have been shown to be conservative in complex models in large sample sizes so these test statistics should be interpreted with caution.

Table S3. Variance Inflation Factors (VIF) per independent variable.

Variable	Category	VIF	1/VIF
**Age**	**41-64**	2.94	0.34
	**65-80**	3.78	0.26
	**81+**	1.64	0.61
**Sex**	**n/a**	2.06	0.49
**Migration status**	**Western migration background**	1.10	0.91
	**Non-western migration background**	1.13	0.89
**Marital status**	**Single**	1.32	0.76
	**Divorced**	1.15	0.87
	**Widowed**	1.39	0.72
**SES Construct**	**Q1, lowest SES**	2.10	0.48
	**Q2**	1.92	0.52
	**Q3**	1.88	0.53
**Physical activity**	**Insufficient**	1.46	0.68
**Body Mass Index (BMI)**	**Underweight (<18,5)**	1.03	0.97
	**Overweight (25-30)**	1.92	0.52
	**Obese (30>)**	1.40	0.71
**Alcohol**	**Regular consumption**	6.11	0.16
**consumption**	**Excessive**	1.59	0.63
**Smoking**	**Former smoker**	2.32	0.43
	**Current smoker**	1.49	0.67
**Loneliness**	**n/a**	2.05	0.49
**Mode of survey completion**	**Internet**	2.29	0.44
	**Face-to-face interview**	1.02	0.98
	**Telephone interview**	1.01	0.99

Table S4. Percentages of adverse health outcomes by SES quartile.

Category	Variables		SES Q1, lowest	SES Q2	SES Q3	SES Q4, highest	p-value
Outcome variables	Chronic disease	At least one	40%	36%	32%	30%	<0.01
	Self-rated health	Bad, fair	34%	26%	21%	18%	<0.01
	Psychological distress	High risk	7%	7%	6%	6%	<0.01
Lifestyle-related factors	Physical activity	Insufficient	35%	32%	34%	35%	<0.01
	Body Mass Index (BMI)	Overweight (BMI:25-30)	38%	36%	35%	33%	<0.01
		Obese (BMI>30)	18%	15%	13%	11%	
	Alcohol consumption	Excessive	7%	8%	7%	7%	<0.01
	Smoking	Former smoker	38%	35%	32%	30%	<0.01
		Current smoker	20%	22%	21%	20%	
Loneliness	Lonely	Some, severe, very severe	48%	44%	41%	40%	<0.01

SES Construct: combination of education, household income quartile and self-reported income adequacy. Based on weighted, multiple-imputed data.

Table S5. Percentages of unhealthy lifestyle behaviors and loneliness by health outcome.

			Chronic disease		Self-rated health		Psychological health		p-value
Category	Variables		none	At least one	(very) good	fair, (very) bad	No or low risk	High risk	
Lifestyle-related factors	Physical activity	Insufficient	33%	37%	31%	44%	33%	50%	<0.01
	Body Mass Index (BMI)	Overweight (BMI:25-30)	34%	40%	34%	37%	35%	32%	<0.01
		Obese (BMI>30)	10%	21%	10%	25%	13%	22%	
	Alcohol consumption	Excessive	7%	7%	7%	7%	7%	8%	<0.01
	Smoking	Former smoker	30%	40%	32%	37%	34%	26%	<0.01
		Current smoker	21%	21%	20%	25%	20%	36%	
Loneliness	Lonely	Some, severe, very severe	37%	53%	36%	65%	40%	87%	<0.01

Based on weighted, multiple-imputed data.

Tables S6-S8 separate SES measures

Table S6. Associations between education, demographic, lifestyle-related factors, loneliness, and health outcomes (n = 445,748).

	OR (95% CI) and % decrease	**Model 1 (SES-model)**	**Model 2 (SES + Lifestyle)**		**Model 3 (SES + loneliness)**		**Model 4 (SES + lifestyle + loneliness)**	
**Chronic Disease**								
Education	primary school	**1.49 (1.46-1.53)**	**1.30 (1.26-1.33)**	39%	**1.38 (1.34-1.41)**	22%	**1.22 (1.19-1.26)**	55%
	lower vocational	**1.27 (1.24-1.29)**	**1.16 (1.14-1.18)**	41%	**1.20 (1.18-1.23)**	26%	**1.11 (1.09-1.13)**	59%
	middle vocational/ secondary	**1.14 (1.12-1.16)**	**1.08 (1.06-1.10)**	43%	**1.11 (1.09-1.13)**	21%	**1.06 (1.04-1.08)**	57%
	Higher vocational/ university	ref	ref		ref		ref	
**Self-rated health**								
Education	primary school	**2.51 (2.43-2.60)**	**1.97 (1.91-2.04)**	36%	**2.14 (2.07-2.21)**	25%	**1.76 (1.70-1.82)**	50%
	lower vocational	**1.92 (1.86-1.97)**	**1.66 (1.61-1.71)**	28%	**1.73 (1.68-1.77)**	21%	**1.53 (1.49-1.58)**	42%
	middle vocational/ secondary	**1.44 (1.40-1.48)**	**1.32 (1.29-1.36)**	27%	**1.36 (1.32-1.40)**	18%	**1.27 (1.23-1.31)**	39%
	Higher vocational/ university	ref	ref		ref		ref	
**Psychological distress**								
Education	primary school	**4.83 (4.39-5.32)**	**3.54 (3.20-3.91)**	34%	**3.43 (3.11-3.78)**	37%	**2.69 (2.43-2.98)**	56%
	lower vocational	**2.60 (2.42-2.79)**	**2.13 (1.98-2.29)**	29%	**1.96 (1.82-2.11)**	40%	**1.68 (1.56-1.81)**	58%
	middle vocational/ secondary	**1.65 (1.54-1.77)**	**1.47 (1.37-1.58)**	28%	**1.41 (1.31-1.52)**	37%	**1.29 (1.19-1.38)**	55%
	Higher vocational/ university	ref	Ref		ref		ref	

Based on weighted, multiple-imputed data. OR's in bold are significant, $p<0.05$

Table S7. Associations between household income quartile, demographic, lifestyle-related factors, loneliness and health outcomes (n = 445,748).

	OR (95% CI) and % decrease	Model 1 (SES-model)	Model 2 (SES + Lifestyle)		Model 3 (SES + loneliness)		Model 4 (SES + lifestyle + loneliness)	
**Chronic Disease**								
Household income quartile	0-25%	**1.56 (1.53-1.59)**	**1.44 (1.41-1.47)**	21%	**1.43 (1.40-1.46)**	23%	**1.34 (1.31-1.37)**	39%
	26-50%	**1.30 (1.28-1.32)**	**1.22 (1.20-1.25)**	27%	**1.23 (1.21-1.26)**	23%	**1.17 (1.15-1.19)**	43%
	51-75%	**1.13 (1.11-1.15)**	**1.10 (1.08-1.12)**	23%	**1.10 (1.08-1.12)**	23%	**1.07 (1.05-1.09)**	46%
	76%-100%	ref	Ref		Ref		ref	
**Self-rated health**								
Household income quartile	0-25%	**2.40 (2.34-2.47)**	**2.07 (2.01-2.12)**	24%	**2.01 (1.96-2.06)**	28%	**1.78 (1.73-1.83)**	44%
	26-50%	**1.85 (1.80-1.89)**	**1.67 (1.63-1.71)**	21%	**1.65 (1.61-1.69)**	24%	**1.52 (1.48-1.55)**	39%
	51-75%	**1.37 (1.34-1.41)**	**1.31 (1.27-1.34)**	16%	**1.30 (1.26-1.33)**	19%	**1.25 (1.22-1.28)**	32%
	76%-100%	ref	ref		Ref		ref	
**Psychological distress**								
Household income quartile	0-25%	**4.64 (4.29-5.02)**	**3.86 (3.57-4.17)**	21%	**2.94 (2.71-3.18)**	47%	**2.53 (2.33-2.74)**	58%
	26-50%	**2.69 (2.49-2.91)**	**2.38 (2.21-2.57)**	18%	**1.96 (1.82-2.12)**	43%	**1.78 (1.65-1.93)**	54%
	51-75%	**1.72 (1.58-1.86)**	**1.61 (1.49-1.75)**	15%	**1.44 (1.33-1.56)**	39%	**1.38 (1.27-1.50)**	47%
	76%-100%	ref	ref		ref		ref	

Based on weighted, multiple-imputed data. OR’s in bold are significant, $p<0.05$

Table S8. Associations between income adequacy, demographic, lifestyle-related factors, loneliness and health outcomes (n = 445,748).

	OR (95% CI) and % decrease	Model 1 (SES-model)	Model 2 (SES + Lifestyle)		Model 3 (SES + loneliness)		Model 4 (SES + lifestyle + loneliness)	
**Chronic disease**								
Income adequacy	Inadequate, major concerns	**2.17 (2.12-2.23)**	**1.96 (1.90-2.01)**	18%	**1.86 (1.81-1.91)**	26%	**1.71 (1.66-1.75)**	39%
	Inadequate, some concerns	**1.66 (1.63-1.69)**	**1.55 (1.52-1.58)**	17%	**1.52 (1.49-1.55)**	21%	**1.44 (1.41-1.47)**	33%
	Adequate, minor concerns	**1.29 (1.26-1.31)**	**1.24 (1.22-1.26)**	17%	**1.24 (1.22-1.26)**	17%	**1.20 (1.19-1.22)**	31%
	Adequate, no concerns	ref	ref		ref		Ref	
**Self-rated health**								
Income adequacy	Inadequate, major concerns	**3.34 (3.24-3.44)**	**2.72 (2.64-2.81)**	26%	**2.42 (2.34-2.49)**	39%	**2.04 (1.98-2.11)**	56%
	Inadequate, some concerns	**2.35 (2.30-2.41)**	**2.08 (2.03-2.13)**	20%	**1.95 (1.90-2.00)**	30%	**1.77 (1.72-1.81)**	43%
	Adequate, minor concerns	**1.56 (1.53-1.59)**	**1.47 (1.44-1.50)**	16%	**1.44 (1.42-1.47)**	21%	**1.38 (1.35-1.40)**	32%
	Adequate, no concerns	ref	ref		ref		ref	
**Psychological distress**								
Income adequacy	Inadequate, major concerns	**14.24 (13.10-15.48)**	**11.45 (10.48-12.51)**	21%	**6.72 (6.15-7.36)**	57%	**5.62 (5.12-6.18)**	65%
	Inadequate, some concerns	**4.82 (4.48-5.18)**	**4.25 (3.95-4.58)**	15%	**2.89 (2.67-3.11)**	51%	**2.62 (2.42-2.84)**	58%
	Adequate, minor concerns	**2.13 (1.99-2.28)**	**2.02 (1.89-2.17)**	10%	**1.68 (1.57-1.80)**	40%	**1.62 (1.51-1.73)**	45%
	Adequate, no concerns	ref	ref		ref		ref	

Based on weighted, multiple-imputed data. OR's in bold are significant, p<0.05

Table S9 SES measures modelled simultaneously

Table S9. Associations between SES, demographic, lifestyle-related factors, loneliness, and health outcomes (n = 445,748).

	OR (95% CI) and % decrease	**Model 1 (SES-model)**	**Model 2 (SES + Lifestyle)**		**Model 3 (SES + loneliness)**		**Model 4 (SES + lifestyle + loneliness)**	
**Chronic Disease**								
Education	primary school	**1.26 (1.23-1.30)**	**1.15 (1.11-1.18)**	42%	**1.22 (1.18-1.25)**	15%	**1.12 (1.08-1.15)**	54%
	lower vocational	**1.13 (1.11-1.15)**	**1.06 (1.04-1.08)**	54%	**1.10 (1.08-1.13)**	23%	**1.05 (1.02-1.07)**	62%
	middle vocational/ secondary	**1.06 (1.04-1.08)**	**1.02 (1.00-1.04)**	67%	**1.05 (1.03-1.07)**	17%	1.01 (0.99-1.03)	83%
	Higher vocational/ university	Ref	Ref		Ref		Ref	
Household income quartile	0-25%	**1.18 (1.15-1.21)**	**1.16 (1.13-1.19)**	11%	**1.15 (1.12-1.18)**	17%	**1.13 (1.10-1.16)**	28%
	26-50%	**1.08 (1.06-1.10)**	**1.06 (1.04-1.08)**	25%	**1.06 (1.04-1.09)**	25%	**1.05 (1.03-1.07)**	38%
	51-75%	**1.03 (1.01-1.05)**	**1.02 (1.00-1.04)**	33%	**1.02 (1.01-1.04)**	33%	1.02 (0.99-1.04)	33%
	76% - 100%	Ref	ref		Ref		Ref	
Income adequacy	Inadequate, major concerns	**1.95 (1.90-2.01)**	**1.82 (1.76-1.87)**	14%	**1.71 (1.66-1.76)**	25%	**1.61 (1.56-1.66)**	36%
	Inadequate, some concerns	**1.54 (1.51-1.57)**	**1.47 (1.44-1.51)**	13%	**1.44 (1.40-1.47)**	19%	**1.38 (1.35-1.41)**	30%
	Adequate, minor concerns	**1.23 (1.21-1.25)**	**1.21 (1.19-1.23)**	9%	**1.20 (1.18-1.22)**	13%	**1.18 (1.16-1.20)**	22%
	Adequate, no concerns	ref	ref		Ref		ref	
**Self-rated health**								
Education	primary school	**1.88 (1.81-1.95)**	**1.59 (1.53-1.65)**	33%	**1.74 (1.68-1.81)**	16%	**1.51 (1.45-1.56)**	42%
	lower vocational	**1.55 (1.51-1.59)**	**1.41 (1.37-1.45)**	25%	**1.48 (1.44-1.52)**	13%	**1.36 (1.32-1.40)**	35%
	middle vocational/ secondary	**1.25 (1.22-1.29)**	**1.19 (1.15-1.22)**	24%	**1.23 (1.19-1.26)**	8%	**1.17 (1.13-1.20)**	32%
	Higher vocational/ university	ref	ref		Ref		Ref	
Household income quartile	0-25%	**1.43 (1.39-1.47)**	**1.38 (1.34-1.42)**	12%	**1.35 (1.31-1.39)**	19%	**1.31 (1.27-1.35)**	28%
	26-50%	**1.27 (1.24-1.31)**	**1.25 (1.21-1.28)**	7%	**1.23 (1.20-1.27)**	15%	**1.21 (1.18-1.24)**	22%
	51-75%	**1.14 (1.11-1.17)**	**1.13 (1.10-1.16)**	7%	**1.12 (1.09-1.15)**	14%	**1.11 (1.08-1.14)**	21%
	76% - 100%	Ref	ref		Ref		Ref	
Income Adequacy	Inadequate, major concerns	**2.61 (2.53-2.70)**	**2.28 (2.20-2.35)**	20%	**1.99 (1.93-2.05)**	39%	**1.78 (1.72-1.83)**	52%
	Inadequate, some concerns	**1.95 (1.90-2.01)**	**1.81 (1.76-1.86)**	15%	**1.68 (1.64-1.72)**	28%	**1.58 (1.54-1.62)**	39%
	Adequate, minor concerns	**1.39 (1.36-1.42)**	**1.35 (1.32-1.38)**	10%	**1.31 (1.29-1.34)**	21%	**1.28 (1.25-1.31)**	28%
	Adequate, no concerns	ref	ref		ref		ref	
**Psychological distress**								
Education	primary school	**3.04 (2.74-3.37)**	**2.49 (2.24-2.77)**	27%	**2.57 (2.31-2.85)**	23%	**2.19 (1.97-2.44)**	42%
	lower vocational	**1.81 (1.68-1.95)**	**1.62 (1.50-1.74)**	23%	**1.55 (1.44-1.68)**	32%	**1.42 (1.31-1.53)**	48%
	middle vocational/ secondary	**1.28 (1.19-1.38)**	**1.20 (1.12-1.29)**	29%	**1.18 (1.10-1.28)**	36%	**1.12 (1.04-1.21)**	57%
	Higher vocational/ university	ref	ref		Ref		Ref	
Household income quartile	0-25%	**1.71 (1.57-1.87)**	**1.63 (1.49-1.78)**	11%	**1.43 (1.31-1.57)**	39%	**1.38 (1.26-1.51)**	46%
	26-50%	**1.39 (1.28-1.51)**	**1.36 (1.25-1.47)**	8%	**1.22 (1.12-1.33)**	44%	**1.20 (1.10-1.31)**	49%
	51-75%	**1.25 (1.15-1.35)**	**1.23 (1.13-1.34)**	8%	**1.15 (1.06-1.25)**	40%	**1.14 (1.05-1.24)**	44%

	76% - 100%	ref	ref		Ref		Ref	
Income Adequacy	Inadequate, major concerns	**10.22 (9.36-11.17)**	**8.92 (8.11-9.76)**	14%	**5.41 (4.93-5.95)**	52%	**4.80 (4.36-5.30)**	59%
	Inadequate, some concerns	**3.75 (3.47-4.04)**	**3.49 (3.23-3.77)**	9%	**2.45 (2.26-2.66)**	47%	**2.32 (2.13-2.52)**	52%
	Adequate, minor concerns	**1.83 (1.71-1.96)**	**1.79 (1.67-1.92)**	5%	**1.52 (1.42-1.64)**	37%	**1.50 (1.39-1.61)**	40%
	Adequate, no concerns	ref	ref		ref		Ref	

Based on weighted, multiple-imputed data. OR’s in bold are significant, $p<0.05$

Tables S10-S13 Subgroup Models

Table S10. Associations between demographic and socio-economic, lifestyle-related factors, loneliness, and health outcomes for males and females.

		OR (95% CI) and % decrease	Model 1 (SES-model)	Model 2 (SES + lifestyle)		Model 3 (SES + loneliness)		Model 4 (SES + lifestyle + loneliness)	
**Male (n = 204,095)**	**Chronic disease**								
	SES Construct	Q1 lowest SES	**1.83 (1.78-1.89)**	**1.67 (1.62-1.72)**	19%	**1.66 (1.61-1.71)**	20%	**1.52 (1.48-1.57)**	37%
		Q2	**1.31 (1.27-1.36)**	**1.24 (1.20-1.28)**	23%	**1.25 (1.21-1.30)**	19%	**1.19 (1.15-1.23)**	39%
		Q3	**1.14 (1.10-1.18)**	**1.10 (1.07-1.14)**	29%	**1.12 (1.08-1.16)**	14%	**1.08 (1.05-1.12)**	43%
		Q4 highest SES	ref	ref		ref		ref	
	**Self-rated health**								
	SES Construct	Q1 lowest SES	**3.42 (3.27-3.56)**	**2.87 (2.75-3.00)**	23%	**2.75 (2.63-2.87)**	28%	**2.36 (2.26-2.47)**	44%
		Q2	**2.06 (1.96-2.15)**	**1.88 (1.79-1.97)**	17%	**1.86 (1.77-1.94)**	19%	**1.71 (1.64-1.79)**	33%
		Q3	**1.48 (1.41-1.56)**	**1.41 (1.34-1.48)**	15%	**1.42 (1.35-1.49)**	13%	**1.35 (1.29-1.42)**	27%
		Q4 highest SES	ref	ref		ref		ref	
	**Psychological distress**								
	SES Construct	Q1 lowest SES	**11.01 (9.48-12.79)**	**9.29 (7.97-1.08)**	17%	**5.98 (5.13-6.98)**	50%	**5.16 (4.41-6.03)**	58%
		Q2	**3.51 (2.97-4.14)**	**3.26 (2.76-3.85)**	10%	**2.48 (2.09-2.93)**	41%	**2.33 (1.97-2.76)**	47%
		Q3	**2.00 (1.68-2.39)**	**1.92 (1.61-2.29)**	8%	**1.74 (1.45-2.09)**	26%	**1.68 (1.40-2.01)**	32%
		Q4 highest SES	ref	ref		ref		ref	
**Female (n = 241,653)**	**Chronic disease**								
	SES Construct	Q1 lowest SES	**1.69 (1.64-1.73)**	**1.50 (1.46-1.54)**	28%	**1.52 (1.48-1.56)**	25%	**1.37 (1.34-1.41)**	46%
		Q2	**1.28 (1.24-1.32)**	**1.20 (1.16-1.23)**	29%	**1.22 (1.18-1.25)**	21%	**1.15 (1.12-1.17)**	46%
		Q3	**1.13 (1.10-1.17)**	**1.09 (1.06-1.12)**	31%	**1.11 (1.07-1.14)**	15%	**1.07 (1.04-1.10)**	46%
		Q4 highest SES	ref	ref		ref		ref	
	**Self-rated health**								
	SES Construct	Q1 lowest SES	**3.12 (3.00-3.24)**	**2.57 (2.47-2.67)**	26%	**2.54 (2.45-2.64)**	27%	**2.16 (2.08-2.25)**	45%
		Q2	**1.95 (1.87-2.03)**	**1.76 (1.69-1.84)**	20%	**1.76 (1.69-1.84)**	20%	**1.62 (1.55-1.69)**	35%
		Q3	**1.43 (1.37-1.49)**	**1.35 (1.30-1.41)**	19%	**1.36 (1.30-1.42)**	16%	**1.30 (1.25-1.36)**	30%
		Q4 highest SES	ref	ref		ref		ref	
	**Psychological distress**								
	SES Construct	Q1 lowest SES	**7.50 (6.65-8.45)**	**5.92 (5.23-6.69)**	24%	**4.06 (3.59-4.61)**	52%	**3.34 (2.93-3.80)**	64%
		Q2	**2.99 (2.65-3.38)**	**2.66 (2.35-3.00)**	17%	**2.11 (1.86-2.39)**	44%	**1.91 (1.69-2.17)**	54%
		Q3	**1.72 (1.51-1.97)**	**1.61 (1.41-1.85)**	15%	**1.45 (1.26-1.66)**	38%	**1.37 (1.19-1.58)**	49%
		Q4 highest SES	ref	ref		ref		ref	

Based on weighted, multiple-imputed data. OR's in bold are significant, $p<0.05$

Table S11. Associations between demographic and socio-economic, lifestyle-related factors, loneliness, and self-rated health for different migration groups.

		OR (95% CI) and % decrease	Model 1 (SES-model)	Model 2 (SES + lifestyle)		Model 3 (SES+ loneliness)		Model 4 (SES + lifestyle + loneliness)	
**Dutch born (n =389,298)**	**Chronic disease**								
	SES Construct	Q1 lowest SES	**1.73 (1.70-1.77)**	**1.56 (1.53-1.59)**	23%	**1.57 (1.54-1.61)**	22%	**1.43 (1.40-1.46)**	41%
		Q2	**1.31 (1.28-1.34)**	**1.23 (1.20-1.26)**	26%	**1.25 (1.22-1.28)**	19%	**1.18 (1.16-1.21)**	42%
		Q3	**1.14 (1.11-1.17)**	**1.10 (1.07-1.13)**	29%	**1.11 (1.09-1.14)**	21%	**1.08 (1.05-1.11)**	43%
		Q4 highest SES	ref	ref		ref		ref	
	**Self-rated health**								
	SES Construct	Q1 lowest SES	**3.18 (3.09-3.28)**	**2.64 (2.56-2.72)**	25%	**2.58 (2.50-2.66)**	28%	**2.19 (2.13-2.26)**	45%
		Q2	**2.00 (1.93-2.06)**	**1.81 (1.75-1.87)**	19%	**1.80 (1.74-1.86)**	20%	**1.65 (1.60-1.71)**	35%
		Q3	**1.44 (1.39-1.49)**	**1.36 (1.32-1.41)**	18%	**1.37 (1.33-1.42)**	16%	**1.31 (1.27-1.36)**	30%
		Q4 highest SES	ref	ref		ref		ref	
	**Psychological distress**								
	SES Construct	Q1 lowest SES	**9.09 (8.25-10.01)**	**7.34 (6.65-8.10)**	22%	**4.91 (4.44-5.42)**	52%	**4.09 (3.69-4.54)**	62%
		Q2	**3.42 (3.09-3.79)**	**3.08 (2.78-3.42)**	14%	**2.41 (2.17-2.68)**	42%	**2.21 (1.98-2.46)**	50%
		Q3	**1.89 (1.69-2.11)**	**1.78 (1.60-1.99)**	12%	**1.61 (1.43-1.80)**	31%	**1.54 (1.37-1.72)**	39%
		Q4 highest SES	ref	ref		Ref		Ref	
**Western migration background (n = 38,445)**	**Chronic disease**								
	SES Construct	Q1 lowest SES	**1.71 (1.60-1.82)**	**1.55 (1.46-1.66)**	23%	**1.54 (1.44-1.64)**	24%	**1.42 (1.33-1.51)**	41%
		Q2	**1.29 (1.20-1.39)**	**1.23 (1.14-1.32)**	21%	**1.22 (1.14-1.31)**	24%	**1.17 (1.09-1.26)**	41%
		Q3	**1.16 (1.08-1.25)**	**1.13 (1.05-1.21)**	19%	**1.13 (1.06-1.22)**	19%	**1.11 (1.03-1.19)**	31%
		Q4 highest SES	ref	ref		ref		ref	
	**Self-rated health**								
	SES Construct	Q1 lowest SES	**3.20 (2.94-3.50)**	**2.69 (2.46-2.94)**	23%	**2.62 (2.40-2.86)**	26%	**2.26 (2.06-2.47)**	43%
		Q2	**1.98 (1.80-2.17)**	**1.82 (1.66-2.00)**	16%	**1.78 (1.63-1.96)**	20%	**1.67 (1.52-1.83)**	32%
		Q3	**1.53 (1.39-1.70)**	**1.47 (1.33-1.63)**	11%	**1.47 (1.33-1.62)**	11%	**1.42 (1.28-1.56)**	42%
		Q4 highest SES	ref	ref		ref		Ref	
	**Psychological distress**								
	SES Construct	Q1 lowest SES	**8.41 (6.35-11.13)**	**6.97 (5.22-9.30)**	19%	**4.82 (3.62-6.42)**	48%	**4.10 (3.06-5.51)**	58%
		Q2	**3.00 (2.22-4.06)**	**2.77 (2.05-3.76)**	12%	**2.16 (1.59-2.92)**	42%	**2.01 (1.48-2.73)**	50%
		Q3	**1.80 (1.30-2.49)**	**1.73 (1.25-2.40)**	9%	**1.64 (1.18-2.27)**	20%	1.59 (1.15-2.20)	26%
		Q4 highest SES	Ref	ref		ref		Ref	
**Non-Western migration background (n = 18,005)**	**Chronic disease**								
	SES Construct	Q1 lowest SES	**1.94 (1.71-2.20)**	**1.79 (1.58-2.03)**	16%	**1.72 (1.51-1.96)**	23%	**1.61 (1.42-1.83)**	35%
		Q2	**1.24 (1.07-1.43)**	**1.20 (1.04-1.38)**	17%	**1.18 (1.02-1.35)**	25%	1.14 (0.99-1.31)	42%
		Q3	1.11 (0.95-1.30)	1.08 (0.93-1.27)	27%	1.08 (0.93-1.26)	27%	1.06 (0.91-1.23)	45%
		Q4 highest SES	Ref	ref		ref		ref	
	**Self-rated health**								
	SES Construct	Q1 lowest SES	**3.73 (3.17-4.39)**	**3.31 (2.81-3.90)**	15%	**3.09 (2.63-3.64)**	23%	**2.79 (2.37-3.28)**	36%
		Q2	**2.13 (1.78-2.56)**	**2.02 (1.69-2.42)**	10%	**1.96 (1.64-2.35)**	15%	**1.87 (1.56-2.24)**	23%
		Q3	**1.54 (1.26-1.87)**	**1.48 (1.22-1.81)**	11%	**1.47 (1.21-1.79)**	13%	**1.42 (1.17-1.73)**	22%

	Q4 highest SES	ref	ref		ref		ref	
**Psychological distress**								
SES Construct	Q1 lowest SES	**7.76 (5.43-11.08)**	**6.54 (4.57-9.37)**	18%	**4.55 (3.15-6.59)**	47%	**3.85 (2.65-5.59)**	58%
	Q2	**2.57 (1.73-3.83)**	**2.39 (1.61-3.56)**	11%	**1.94 (1.29-2.93)**	40%	**1.81 (1.20-2.72)**	48%
	Q3	**1.63 (1.05-2.53)**	1.53 (0.99-2.38)	16%	1.39 (0.88-2.19)	38%	1.28 (0.81-2.03)	56%
	Q4 highest SES	ref	ref		ref		ref	

Based on weighted, multiple-imputed data. OR’s in bold are significant, $p<0.05$

Table S12. Associations between demographic and socio-economic, lifestyle-related factors, loneliness and health outcomes for different marital status groups.

**Married (n = 316,000)**	**Chronic disease**	OR (95% CI) and % decrease	**Model 1 (SES-model)**	**Model 2 (SES + lifestyle)**		**Model 3 (SES+ loneliness)**		**Model 4 (SES + lifestyle + loneliness)**	
	SES Construct	Q1 lowest SES	**1.67 (1.64-1.72)**	**1.49 (1.46-1.53)**	27%	**1.53 (1.49-1.56)**	21%	**1.38 (1.34-1.41)**	43%
		Q2	**1.29 (1.26-1.32)**	**1.20 (1.17-1.23)**	31%	**1.23 (1.20-1.26)**	21%	**1.16 (1.13-1.18)**	45%
		Q3	**1.14 (1.11-1.17)**	**1.09 (1.07-1.12)**	36%	**1.11 (1.09-1.14)**	21%	**1.07 (1.05-1.10)**	50%
		Q4 highest SES	Ref	Ref		Ref		Ref	
	**Self-rated health**	Q1 lowest SES	**3.26 (3.15-3.27)**	**2.67 (2.58-2.76)**	26%	**2.65 (2.56-2.74)**	27%	**2.23 (2.15-2.31)**	46%
	SES Construct	Q2	**2.03 (1.96-2.11)**	**1.82 (1.76-1.89)**	20%	**1.83 (1.76-1.89)**	19%	**1.66 (1.61-1.72)**	36%
		Q3	**1.46 (1.40-1.52)**	**1.37 (1.32-1.43)**	20%	**1.39 (1.34-1.44)**	15%	**1.32 (1.27-1.37)**	30%
		Q4 highest SES	Ref	Ref		Ref		Ref	
**Single (n = 46,625)**	**Chronic disease**	Q1 lowest SES	**2.10 (1.96-2.25)**	**1.98 (1.85-2.11)**	11%	**1.84 (1.72-1.97)**	24%	**1.75 (1.64-1.88)**	32%
	SES Construct	Q2	**1.39 (1.28-1.50)**	**1.35 (1.25-1.46)**	10%	**1.31 (1.21-1.42)**	21%	**1.28 (1.18-1.39)**	28%
		Q3	**1.17 (1.08-1.26)**	**1.14 (1.06-1.24)**	18%	**1.14 (1.06-1.23)**	18%	**1.12 (1.04-1.21)**	29%
		Q4 highest SES	Ref	Ref		Ref		Ref	
	**Self-rated health**	Q1 lowest SES	**3.66 (3.35-4.00)**	**3.17 (2.90-3.46)**	18%	**2.83 (2.59-3.10)**	31%	**2.51 (2.30-2.75)**	43%
	SES Construct	Q2	**2.06 (1.86-2.28)**	**1.94 (1.75-2.14)**	11%	**1.84 (1.66-2.03)**	21%	**1.74 (1.58-1.92)**	30%
		Q3	**1.48 (1.33-1.64)**	**1.42 (1.28-1.57)**	13%	**1.41 (1.27-1.57)**	15%	**1.37 (1.23-1.51)**	23%
		Q4 highest SES	Ref	Ref		Ref		Ref	
**Divorced (n = 30,927)**	**Chronic disease**	Q1 lowest SES	**1.95 (1.81-2.10)**	**1.76 (1.63-1.90)**	20%	**1.72 (1.60-1.86)**	24%	**1.58 (1.46-1.70)**	39%
	SES Construct	Q2	**1.31 (1.20-1.42)**	**1.25 (1.15-1.36)**	19%	**1.24 (1.14-1.35)**	23%	**1.19 (1.10-1.30)**	39%
		Q3	**1.15 (1.05-1.27)**	**1.13 (1.02-1.25)**	13%	**1.13 (1.03-1.25)**	13%	**1.11 (1.01-1.22)**	27%
		Q4 highest SES	Ref	Ref		Ref		Ref	
	**Self-rated health**	Q1 lowest SES	**3.21 (2.88-3.57)**	**2.78 (2.50-3.10)**	19%	**2.57 (2.31-2.86)**	29%	**2.28 (2.05-2.54)**	42%
	SES Construct	Q2	**1.81 (1.60-2.04)**	**1.71 (1.52-1.93)**	12%	**1.64 (1.46-1.85)**	21%	**1.57 (1.40-1.77)**	30%
		Q3	**1.39 (1.21-1.58)**	**1.35 (1.19-1.54)**	10%	**1.34 (1.18-1.52)**	13%	**1.31 (1.15-1.48)**	21%
		Q4 highest SES	Ref	Ref		Ref		Ref	
**Widowed (n = 52,556)**	**Chronic disease**	Q1 lowest SES	**1.41 (1.35-1.48)**	**1.27 (1.21-1.34)**	34%	**1.32 (1.26-1.38)**	22%	**1.20 (1.07-1.26)**	51%
	SES Construct	Q2	**1.16 (1.10-1.22)**	**1.09 (1.04-1.15)**	44%	**1.12 (1.07-1.18)**	25%	**1.06 (1.04-1.10)**	62%
		Q3	1.02 (0.97-1.09)	1.00 (0.95-1.06)		1.01 (0.95-1.07)		0.99 (0.93-1.04)	
		Q4 highest SES	Ref	Ref		Ref		Ref	
	**Self-rated health**	Q1 lowest SES	**2.16 (2.02-2.30)**	**1.83 (1.71-1.96)**	28%	**1.91 (1.79-2.04)**	22%	**1.66 (1.55-1.77)**	43%
	SES Construct	Q2	**1.57 (1.47-1.68)**	**1.43 (1.34-1.53)**	25%	**1.48 (1.39-1.59)**	16%	**1.36 (1.28-1.47)**	37%
		Q3	**1.26 (1.17-1.36)**	**1.21 (1.12-1.31)**	19%	**1.22 (1.13-1.31)**	15%	**1.18 (1.09-1.27)**	31%
		Q4 highest SES	ref	ref		ref		ref	

Based on weighted, multiple-imputed data. OR's in bold are significant, $p<0.05$

Table S13. Associations between demographic and socio-economic, lifestyle-related factors, loneliness, and health outcomes for different age groups.

		OR (95% CI) and % decrease	**Model 1 (SES-model)**	**Model 2 (SES + lifestyle)**		**Model 3 (SES + loneliness)**		**Model 4 (SES + lifestyle + loneliness)**	
**Age 19-40**	**Chronic disease**								
**(n = 68,434)**	SES Construct	Q1 lowest SES	**2.14 (2.01-2.27)**	**1.99 (1.87-2.13)**	13%	**1.83 (1.72-1.95)**	27%	**1.73 (1.63-1.85)**	37%
		Q2	**1.39 (1.30-1.48)**	**1.34 (1.25-1.43)**	13%	**1.29 (1.20-1.37)**	26%	**1.25 (1.17-1.33)**	36%
		Q3	**1.16 (1.08-1.24)**	**1.13 (1.06-1.21)**	19%	**1.12 (1.05-1.20)**	25%	**1.10 (1.03-1.17)**	37%
		Q4 highest SES	Ref	Ref		Ref		Ref	
	**Self-rated health**								
	SES Construct	Q1 lowest SES	**4.10 (3.75-4.48)**	**3.45 (3.15-3.79)**	21%	**2.95 (2.70-3.23)**	37%	**2.56 (2.33-2.81)**	50%
		Q2	**2.21 (2.01-2.43)**	**2.03 (1.84-2.24)**	15%	**1.88 (1.71-2.06)**	27%	**1.75 (1.59-1.93)**	38%
		Q3	**1.53 (1.37-1.70)**	**1.44 (1.30-1.60)**	17%	**1.43 (1.29-1.58)**	19%	**1.36 (1.23-1.51)**	32%
		Q4 highest SES	Ref	Ref		Ref		Ref	
	**Psychological distress**								
	SES Construct	Q1 lowest SES	**7.49 (6.41-8.75)**	**6.20 (5.28-7.28)**	20%	**3.92 (3.32-4.61)**	55%	**3.24 (2.74-3.84)**	65%
		Q2	**3.02 (2.54-3.59)**	**2.74 (2.30-3.26)**	14%	**2.06 (1.73-2.47)**	48%	**1.86 (1.56-2.23)**	57%
		Q3	**1.77 (1.49-2.12)**	**1.67 (1.40-1.99)**	13%	**1.49 (1.24-1.79)**	36%	**1.41 (1.17-1.69)**	47%
		Q4 highest SES	Ref	Ref		Ref		Ref	
**Age 41-64**	**Chronic disease**	Q1 lowest SES	**1.94 (1.88-2.00)**	**1.71 (1.66-1.77)**	24%	**1.73 (1.68-1.79)**	22%	**1.55 (1.50-1.60)**	41%
**(n = 142,790)**	SES Construct	Q2	**1.35 (1.31-1.40)**	**1.26 (1.22-1.30)**	26%	**1.29 (1.25-1.33)**	17%	**1.21 (1.17-1.24)**	40%
		Q3	**1.17 (1.14-1.21)**	**1.12 (1.09-1.16)**	29%	**1.15 (1.11-1.19)**	12%	**1.10 (1.07-1.14)**	41%
		Q4 highest SES	Ref	Ref		Ref		Ref	
	**Self-rated health**								
	SES Construct	Q1 lowest SES	**3.73 (3.57-3.89)**	**3.07 (2.94-3.21)**	24%	**2.96 (2.83-3.09)**	28%	**2.51 (2.40-2.62)**	45%
		Q2	**2.10 (2.00-2.20)**	**1.89 (1.81-2.00)**	19%	**1.90 (1.81-1.99)**	18%	**1.74 (1.66-1.82)**	33%
		Q3	**1.49 (1.42-1.56)**	**1.41 (1.34-1.47)**	16%	**1.43 (1.36-1.50)**	12%	**1.36 (1.30-1.42)**	27%
		Q4 highest SES	Ref	Ref		Ref		Ref	
	**Psychological distress**								
	SES Construct	Q1 lowest SES	**10.91 (9.56-12.45)**	**8.76 (7.64-10.04)**	22%	**5.83 (5.09-6.67)**	51%	**4.89 (4.25-5.62)**	61%
		Q2	**3.52 (3.06-4.06)**	**3.18 (2.75-3.68)**	13%	**2.52 (2.18-2.91)**	40%	**2.34 (2.02-2.71)**	47%
		Q3	**1.90 (1.64-2.21)**	**1.80 (1.55-2.09)**	11%	**1.64 (1.41-1.90)**	29%	**1.57 (1.35-1.82)**	37%
		Q4 highest SES	Ref	Ref		Ref		Ref	
**Age 65-80**	**Chronic disease**								
**(n = 192,640)**	SES Construct	Q1 lowest SES	**1.34 (1.32-1.37)**	**1.20 (1.17-1.22)**	41%	**1.27 (1.25-1.30)**	21%	**1.15 (1.12-1.17)**	56%
		Q2	**1.11 (1.08-1.13)**	**1.04 (1.02-1.06)**	64%	**1.08 (1,06-1,10)**	27%	1.02 (0.99-1.04)	
		Q3	**1.03 (1.01-1.05)**	1.00 (0.98-1.02)		1.01 (0.99-1.04)		0.99 (0.97-1.01)	
		Q4 highest SES	Ref	Ref		Ref		Ref	
	**Self-rated health**								
	SES Construct	Q1 lowest SES	**2.39 (2.32-2.46)**	**1.95 (1.89-2.01)**	32%	**2.11 (2,05-2.18)**	20%	**1.77 (1.72-1.82)**	45%

		Q2	**1.65 (1.60-1.70)**	**1.48 (1.43-1.53)**	26%	**1.56 (1.51-1.61)**	14%	**1.42 (1.37-1.46)**	35%
		Q3	**1.29 (1.25-1.34)**	**1.23 (1.19-1.27)**	21%	**1.25 (1.21-1.29)**	14%	**1.20 (1.16-1.24)**	31%
		Q4 highest SES	Ref	Ref		Ref		Ref	
	**Psychological distress**								
	SES Construct	Q1 lowest SES	**7.98 (6.92-9.21)**	**5.75 (4.96-6.66)**	32%	**5.18 (4.48-5.98)**	40%	**3.93 (3.38-4.55)**	58%
		Q2	**2.93 (2.52-3.41)**	**2.47 (2.12-2.88)**	24%	**2.32 (2.00-2.70)**	32%	**2.01 (1.72-2.34)**	48%
		Q3	**1.91 (1.62-2.25)**	**1.78 (1.50-2.10)**	14%	**1.66 (1.40-1.96)**	27%	**1.55 (1.31-1.84)**	40%
		Q4 highest SES	ref	ref		ref		Ref	
**Age 81+ (n = 41,884)**	**Chronic disease**								
	SES Construct	Q1 lowest SES	**1.14 (1.10-1.19)**	**1.06 (1.02-1.10)**	57%	**1.10 (1.06-1.15)**	29%	1.03 (0.99-1.07)	79%
		Q2	1.02 (0.98-1.06)	0.97 (0.93-1.01)		1.00 (0.96-1.04)		**0.96 (0.92-0.99)**	
		Q3	0.99 (0.96-1.04)	0.98 (0.94-1.02)		0.99 (0.95-1.03)		0.97 (0.93-1.01)	
		Q4 highest SES	Ref	Ref		Ref		Ref	
	**Self-rated health**								
	SES Construct	Q1 lowest SES	**1.63 (1.55-1.72)**	**1.44 (1.37-1.51)**	30%	**1.53 (1.46-1.61)**	16%	**1.37 (1.30-1.44)**	41%
		Q2	**1.32 (1.25-1.39)**	**1.22 (1.16-1.29)**	31%	**1.29 (1.22-1.35)**	9%	**1.20 (1.14-1.26)**	38%
		Q3	**1.18 (1.11-1.26)**	**1.14 (1.08-1.21)**	22%	**1.17 (1.10-1.23)**	6%	**1.13 (1.07-1.19)**	28%
		Q4 highest SES	Ref	Ref		Ref		Ref	
	**Psychological distress**								
	SES Construct	Q1 lowest SES	**6.39 (5.02-8.13)**	**4.97 (3.88-6.36)**	26%	**4.95 (3.86-6.33)**	27%	**3.91 (3.03-5.04)**	46%
		Q2	**3.11 (2.43-3.99)**	**2.64 (2.05-3.39)**	22%	**2.72 (2.11-3.51)**	18%	**2.31 (1.79-3.00)**	38%
		Q3	**1.82 (1.35-2.46)**	**1.68 (1.24-2.28)**	17%	**1.65 (1.22-2.23)**	21%	**1.51 (1.11-2.05)**	38%
		Q4 highest SES	ref	ref		ref		Ref	

Based on weighted, multiple-imputed data. OR's in bold are significant, $p<0.05$
